# Subcellular Localization Relevance and Cancer-Associated Mechanisms of Diacylglycerol Kinases

**DOI:** 10.3390/ijms21155297

**Published:** 2020-07-26

**Authors:** Antonietta Fazio, Eric Owusu Obeng, Isabella Rusciano, Maria Vittoria Marvi, Matteo Zoli, Sara Mongiorgi, Giulia Ramazzotti, Matilde Yung Follo, James A. McCubrey, Lucio Cocco, Lucia Manzoli, Stefano Ratti

**Affiliations:** 1 Cellular Signalling Laboratory, Department of Biomedical and Neuromotor Sciences (DIBINEM), University of Bologna, Via Irnerio 48, 40126 Bologna, Italy; antonietta.fazio2@unibo.it (A.F.); eric.owusuobeng2@unibo.it (E.O.O.); isabella.rusciano3@unibo.it (I.R.); mariavittoria.marvi2@unibo.it (M.V.M.); s.mongiorgi@unibo.it (S.M.); giulia.ramazzotti@unibo.it (G.R.); matilde.follo@unibo.it (M.Y.F.); lucio.cocco@unibo.it (L.C.); stefano.ratti@unibo.it (S.R.); 2 Center for the Diagnosis and Treatment of Hypothalamic-Pituitary Diseases-Pituitary Unit, IRCCS Istituto delle Scienze Neurologiche di Bologna (Institute of Neurological Sciences of Bologna), 40126 Bologna, Italy; matteo.zoli4@unibo.it; 3 Department of Biomedical and Neuromotor Sciences (DIBINEM), University of Bologna, 40126 Bologna, Italy; 4 Department of Microbiology and Immunology, Brody School of Medicine at East Carolina University, Greenville, NC 27858, USA; MCCUBREYJ@ecu.edu

**Keywords:** diacylglycerol, phosphoinositide, PI3K/Akt/mTOR, DGKs, lipids, cancer

## Abstract

An increasing number of reports suggests a significant involvement of the phosphoinositide (PI) cycle in cancer development and progression. Diacylglycerol kinases (DGKs) are very active in the PI cycle. They are a family of ten members that convert diacylglycerol (DAG) into phosphatidic acid (PA), two-second messengers with versatile cellular functions. Notably, some DGK isoforms, such as DGKα, have been reported to possess promising therapeutic potential in cancer therapy. However, further studies are needed in order to better comprehend their involvement in cancer. In this review, we highlight that DGKs are an essential component of the PI cycle that localize within several subcellular compartments, including the nucleus and plasma membrane, together with their PI substrates and that they are involved in mediating major cancer cell mechanisms such as growth and metastasis. DGKs control cancer cell survival, proliferation, and angiogenesis by regulating Akt/mTOR and MAPK/ERK pathways. In addition, some DGKs control cancer cell migration by regulating the activities of the Rho GTPases Rac1 and RhoA.

## 1. Introduction

Phosphoinositides (PIs) represent a tiny component of the total phospholipid content in eukaryotic cell membranes, but they regulate numerous cellular activities such as cell adhesion [1], migration [2], apoptosis [3], vesicular trafficking [4], and post-translational modifications [5]. These processes are consistent with cancer-associated cellular mechanisms. PI metabolism is controlled by several kinases, phosphatases, and phospholipases following their stimulation by different external stimuli. An increasing number of studies report that alterations in the PI cycle, resulting from dysfunctional PI metabolic enzymes, are involved in cancer [6,7,8].

Diacylglycerol kinases (DGKs) are a family of ten PI metabolic kinases (α, β, γ, δ, ε, ζ, η, θ, ι, and κ) that participate in the PI cycle by catalyzing the phosphorylation of diacylglycerol (DAG) to generate phosphatidic acid (PA), hence, regulating these two-second messengers [9]. DAG and PA are involved in the regulation of several critical enzymes, including mammalian target of rapamycin (mTOR) [10], phosphatidylinositol-4-phosphate 5-kinase (PIP5K), the Ras GTPase-activating protein (GAP), rapidly accelerated fibrosarcoma-1 (Raf-1) kinase, protein kinases C (PKCs), mammalian uncoordinated 13 (Unc-13), Chimaerins (which activate Rac GTPase), and rat sarcoma virus guanyl nucleotide-releasing protein (RasGRP), which are involved in cell proliferation and migration [11,12]. Although DGKs can utilize different molecular DAG species independent of PI turnover pathways [13], the conversion of DAG to PA by DGKs represents one of the first steps in PI resynthesis, where phosphatidylinositol 4,5 biphosphate (PtdIns(4,5)P_2_) levels are replenished [14]. As such, DGK signaling is pivotal in modulating the balance between these two essential bioactive lipids, DAG, and PA. 

Accumulating evidence demonstrates that DGKs, as well as phospholipases C (PLCs) and PKCs, are distributed across several subcellular compartments together with their substrates [15,16,17] and, as PLCs and PKCs, they are involved in cell regulation [18,19]. Nuclear localization allows DGKs to participate in a PI cycle which is independent of that of the plasma membrane [20,21]. Therefore, DGK activity may regulate distinct cellular functions and explain the complexities that surround DGK signaling [17]. In fact, DGKs regulate cytokine/growth factor-mediated cell proliferation and migration, cell growth, seizure activity, and insulin receptor-mediated glucose metabolism, suggesting that DGKs may also be involved in several diseases including epilepsy and diabetes [22,23]. 

Of particular note is the involvement of DGKs in cancer development and progression [24,25,26]. For instance, mutations in the DGKα gene can drive pancreatic cancer [24] as well as promote hepatocellular carcinoma (HCC) progression by activating the mitogen-activated protein kinase (MAPK) pathway [27]. In 3D colon and breast cancer models, DGKα was reported to promote cell survival by regulating Src [28]. For these reasons, there are several reports suggesting that DGKα may be a promising therapeutic target in cancer therapy [7,29]. Meanwhile, in colorectal cancer (CRC), DGKγ plays tumor-suppressive roles, while DGKζ activity promotes tumor progression [26,30,31]. Moreover, DGKη and DGKδ regulate cell growth and proliferation in cervical cancer cell lines [32,33], whereas epigenetic changes in DGKι are reported in glioblastoma and HCC cells [34,35]. Despite the numerous reports of the involvement of DGKs in cancer as well as their clinical potential, the comprehension of the specific cellular functions regulated by DGKs in cancer is not yet complete. This review aims to provide up-to-date knowledge of the regulatory roles played by DGKs in cancer cell survival and metastasis, while also highlighting the downstream regulation of DGKs, the role of their cellular localization and summing up current knowledge on targeting DGKs in cancer therapies.

## 2. Activation and Regulation of DGK Isozymes

The 10 members of the mammalian DGK family are classified into 5 different subtypes depending on their structural motifs [36]: type I (DGKs α, β, and γ), type II (DGKs δ, η, and κ), type III (DGKε), type IV (DGKs ζ, and ι) and type V (DGKθ). Moreover, some DGK isoforms undergo further alternative splicing which often changes either the distribution or activity of the enzyme [37,38]. Their differences may be attributed to their evolution, in order to regulate specific cellular processes evident in higher vertebrates [39]. DGKs are ubiquitous kinases that are mainly expressed in the brain and hemopoietic tissue [40]. They are prominently distributed across several different regions of the brain, including the cerebellum, hippocampus, and the olfactory bulb, therefore suggesting involvement of DGKs in central nervous system functions [41]. Some DGK isoforms are also expressed in the retina (ε, γ, ι) [41], in striated (δ and ζ) and cardiac muscle (β and ε) [41,42] and in the lungs (α, ε, ζ and η) [43]. So far, it has been shown that different DGK isoforms can be co-expressed in the same tissue and even in the same cell, suggesting that each subtype may carry out tissue or cell-specific functions [41].

Until now, all recognized mammalian DGKs possess two common kinase domains comprising a conserved catalytic domain, which is characterized by a highly conserved ATP binding site and an accessory domain [44]. The possession of additional distinct domains, that seems to have regulatory roles, as shown in DGK family types, contributes to isoform-specific functions and diversity among mammalian DGK isoforms [17]. For instance, type I isoforms (α, β, and γ) participate in calcium (Ca^2+^) signaling because of their Ca^2+^ binding motif, while the carboxyl terminus-based sterile alpha motif (SAM) of DGKδ, which is a type II DGK, promotes protein-protein interactions [45]. In addition, DGKδ possesses a PH domain that weakly binds to phosphatidylinositols [46]. The nuclear localization of type IV DGKs (ζ and ι) is enhanced via their nuclear localization sequence (NLS). This domain also serves as substrate for conventional PKCs and is homologous to the phosphorylation domain of the myristoylated alanine-rich kinase substrate (MARCKS) protein. DGKθ, which is the only type V DGK isoform, can be differentiated by its PH domain, three C1 domains, and a Ras-associating domain that mediates Ras signaling [47].

All DGKs possess at least two cysteine-rich regions similar to the DAG-binding C1A and C1B domains of PKC [48]. The C1 regions of DGKs allow membrane binding either through protein interactions, as demonstrated by several DGKs and β-arrestin [49] or through lipid interactions, as shown by DGKα and the lipid product of phosphoinositide 3-kinase (PI3K) [50,51]. C1 domains are recognized phorbol ester or DAG binding regions, but several studies have tried to evaluate the binding potential of the C1 domains of some DGKs to a DAG analog or phorbol ester reported that only the C1A domain of DGK β and γ displays successful binding [52]. Therefore, it is not clear whether the C1 domains of all DGKs can actually bind DAG. 

The activation and regulation of DGKs is a very complex process that needs further studies to be fully comprehended [37]. Given the structural and subcellular localization differences, it may be possible that different activation mechanisms exist for each individual DGK isoform. Considering also the ability of DGKs to translocate to different cellular sites, the presence of post-translational modifications and their binding to different cofactors, such as membrane lipids and Ca^2+^, may produce some degree of diversity in their functions. DAG is accessed by DGKs upon their translocation to DAG-producing cellular membranes, where DGKs are proposed to be activated during agonist or kinase promoted phosphorylation or following their binding to some cofactors or to other proteins [39]. Indeed, some studies reported that the distinct activities observed in the various DGK isoforms may depend on the type of agonist and the cofactors they bind to during their activation [53]. For example, DGKα, which is one of the most studied DGK isoforms, demonstrates this complexity in T lymphocytes. Based on the type of agonist used to activate DGKα in T cells, it translocates to two different membrane compartments: stimulation with interleukin 2 (IL-2) induces the translocation of DGKα from the cytosol to the perinuclear region [54], whereas the translocation from the cytosol to the plasma membrane occurs when DGKα is stimulated by T-cell antigen receptor [55]. Several different cofactors, such as Ca^2+^, which binds to the EF-hand motif and membrane lipids, including phosphatidylserine (PS), sphingosine, the PI3K lipid products, PtdIns(3,4)P_2_, and PtdIns(3,4,5)P_3_ have been reported to modify DGKα activity both in vitro and in vivo [56]. As for other DGK isoforms, activation of DGKδ may be enhanced by the binding of its PH domain to phosphatidylinositols [41], DGKε is inhibited by both PtdIns(4,5)P_2_ and PS, while DGKζ is activated by both PtdIns(4,5)P_2_ and PS [57]. Moreover, the protein-protein interaction between DGKθ and RhoA is involved in the regulation of the activity of DGKθ, where its kinase activity is completely reduced by RhoA [17]. 

Several studies showed that the specificity of DGK activities could also be attributed to its association with or inhibition of DAG-activated proteins, such as the RasGRP proteins [58]. Generally, when DAG is abundant, RasGRPs activate either Rap or Ras, or both, and this mechanism is RasGRP-isoform specific. Consequently, the downstream effects of DGKs diverge because DGK isozymes bind to different isoforms of RasGRP [59]. Hence, the functional specificity of DGKs depends on their interactions. For example, type IV DGKs, ζ, and ι, are all structurally similar, but they induce opposing effects on Ras signaling. DGKζ attenuates Ras signaling both in vitro [58,60] and in vivo [60], whereas DGKι enhances it [59]. These opposing effects were mainly dependent on the ability of DGKs ζ and ι to bind and inhibit specific RasGRP enzymes, respectively RasGRP1 and RasGRP3 [58,59]. Since the activities of DGKs maintain a balance between DAG and PA levels, DGKs can also be associated with proteins whose activities are regulated by PA. In fact, DGKs regulate either directly or indirectly Rac1 [30], mTOR [10], PIP5K type 1α [17], and atypical PKCs [50], all regulated by PA while mediating several essential cellular effects such as cell survival, migration, and vesicle trafficking [11,12].

DGKs were initially described as modulators of the classical and novel PKC family members. However, some DGKs form complexes with certain DAG-sensitive PKC isoforms, thus being regulated by PKC-dependent phosphorylation [36,50]. Indeed, these DGKs and their respective PKC counterparts mutually regulate each other’s enzyme activities, as seen in the case of DGKζ and PKCα. At immune and nervous synapses, the activation of DGKζ and PKCα is mutually regulated by both kinases [50]. At basal conditions, DGKζ phosphorylates DAG and prevents PKCα activation [61] but upon stimulation, there is an overproduction of local DAG levels that is too abundant for DGKζ to phosphorylate, leading to the availability of excess DAG. Consequently, high levels of DAG activate PKCα, which phosphorylates DGKζ, causing their physical dissociation [61] and promoting transient or even prolonged activation of PKCα. In the context of cancer, the DGKζ-PKCα axis could be important in regulating signaling in tumor cells. For instance, DGKζ undergoes a PKCα-dependent phosphorylation to enhance its shuttle from the nucleus to the cytosol [62], a biological process which is implicated in cancer cells responding to stress conditions [63]. Moreover, DGKα is involved in inflammation in tumor cells by positively regulating tumor necrosis factor α (TNFα)-induced nuclear factor kappa-light-chain-enhancer of activated B cells (NF-κB) activation via a PKCζ-mediated Ser311 phosphorylation of the NF-κB subunit p65/RelA [64].

Overall, DGKs can be activated by several mechanisms which might be isoform-specific, and they produce different cellular outcomes based on the type of co-factors and proteins they associate with. The concept of DGK-isoform specific functions is supported by mouse knockout studies showing that targeted deletion of specific DGK isoforms leads to a distinct phenotype [59,65,66,67,68]. 

## 3. Cellular Localization and Distribution of DGKs

As previously reported, DGKs can translocate to various distinct cellular compartments depending on the type of agonist. This supports the fact that the activities of DGKs may be confined to specific DAG pools produced after receptor activation [17]. This is the case of DGKε, which only phosphorylates DAG species that possess an arachidonoyl group at the *sn*-2 position [50]. Moreover, recent studies have also shown the presence of an alternative DAG metabolic pathway where individual DGK isoforms phosphorylate different molecular DAG pools or species independently from PI turnover [13]. For instance, DGKδ2 interacts via its SAM domain with the ER enzyme sphingomyelin synthase related protein (SMSr) generating DAG from phosphatidylethanolamine and ceramide [69]. Through a flip-flop mechanism, DAG produced by SMSr in the ER crosses the ER membrane from the lumen region to the cytosolic region to give access to DGKδ2 [69]. A previous work by van der Bend and colleagues showed that receptor activation of DGK in cells induces a physiological DAG generation while treating cells with exogenous PLC induces a global, non-specific DAG production [17,70]. Moreover, receptor activation of DGKs exhibited a significant increase in kinase activity, compared to the kinase activity produced from treating cells with exogenous PLC. Following this observation, the authors suggested that DGKs are activated only in spatially restricted subcellular sites characterized by DAG production because DGKs cannot use DAG pools that are randomly generated in the plasma membrane [17,70]. 

DGKs usually localize within several cell compartments, with majority localizing at least partially within the plasma membrane. This is either constitutively, as seen in the case of DGKκ [71], or following stimulation with specific agonists, such as DGKδ1, which is translocated to the plasma membrane upon exposure to phorbol esters [72], or DGKα, following engagement of the T cell receptor [55]. Moreover, DGKs θ and ζ are found at the plasma membrane upon activation of some G protein-coupled receptors (Table 1) [73,74]. Several studies showed that the nuclear inositide signaling is involved in regulating essential cellular processes, such as cell cycle and differentiation [75,76,77,78], while it is also implicated in several pathologies, including myelodysplastic syndromes, brain diseases, and cancer [79,80,81,82]. Interestingly, DGKs, which have been discovered in nearly all cell compartments, were also found in the nucleus. Notably, DGKs play a critical role in the PI cycle, and the conversion of DAG to PA by DGKs represents the first step in resynthesizing PIs [14]. In addition, different agonists, such as insulin-like growth factor 1 (IGF-1) or thrombin, can generate DAG in the nucleus but not in the plasma membrane. Moreover, nuclear DAG levels fluctuate independently of extranuclear DAG during cell cycle [20]. Among the nuclear DGKs, DGK α, ζ, and ι translocate in and out of the nucleus [17], while DGKγ is shuttled from the cytoplasm to the nucleus [83] and a significant fraction of DGKθ localize mainly within nuclear speckles [84,85]. DGKθ can also translocate from the cytosol to the plasma membrane upon stimulation by PKCs and activated GPCRs [86]. In addition, nuclear DGKs ζ and ι localize within distinct nuclear compartments [17,84] whereas DGKα localizes mainly at the nuclear periphery [17,71].

DGKs also localize within other organelles and this may be cell type-specific [87,88]. Localization and expression of DGKs in the brain remain elusive. However, DGKε localizes to the subsurface cisterns of cerebellar Purkinje cells and colocalizes with inositol trisphosphate receptor-1 (InsP_3_R-1) in dendrites and axons of the brain, thus confirming the involvement of DGKε in neuronal and brain functions [89]. Moreover, DGKε can also localize within the endoplasmic reticulum and the plasma membrane [87]. In adrenal cells, a group of PI signaling molecules are expressed significantly in zona glomerulosa cells and medullary chromaffin cells in the adrenal gland. The same study showed that DGKγ localizes to the Golgi complex, DGKε to the plasma membrane, and DGKζ to the nucleus of adrenal cells [88]. Furthermore, DGKs δ and η localize to endosomes [37,45].

Even though the specific functions of DGKs are not clearly known yet, several reports demonstrated the presence of DGK activity in cellular contents containing cytoskeletal components and the possible involvement of DGKs in cytoskeletal remodeling and cellular morphology [90,91]. DGKs can interact with proteins associated with cytoskeletal reorganization, such as Rac and Rho GTPases, PIP5K, Cdc42, and Rho-GDI [17,91,92]. For example, DGKζ binds directly to Rac1 to form a complex with Rho-GDI and PAK1 [93], DGKβ co-localized with actin filaments [94], whereas endogenous DGKζ co-purified with cytoskeletal proteins and localized to the leading edge of C2 myoblasts [42] and glioblastoma cells [58].

## 4. The Impact of DGKs in the Regulation of Cancer Cell Mechanisms

**Figure 1:**
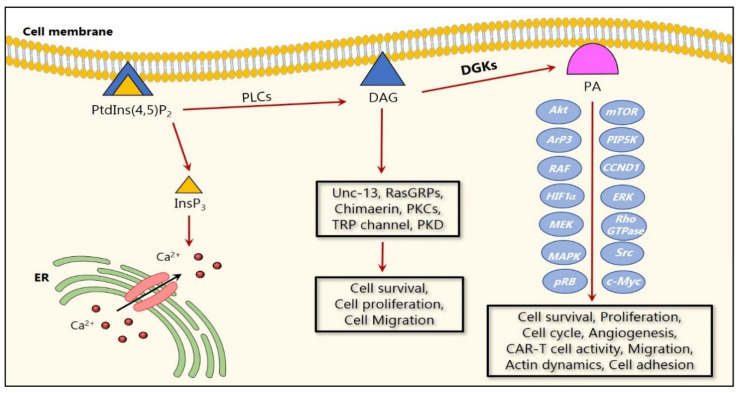
Cartoon representation of DGK signaling in cancer. Upon their activation, DGKs phosphorylate DAG to form PA, which results in a series of signaling events resulting from the alterations in the signaling of several molecular targets. Several studies reported that DGKs regulate cancer cell survival and proliferation via MAPK and Akt/mTOR pathways. In addition, DGKs regulate migration via the Ras/RAF/MEK/ERK pathway and the Rho GTPase family members. On the other hand, DAG activates several critical proteins including both conventional and novel PKCs, mammalian Unc-13, chimaerins which activate Rac GTPase, and RasGRPs, which are involved in cell proliferation and migration.

Despite all the progress made in medicine, cancer remains one of the frequently occurring causes of death in humans. Therefore, it is important to better understand the various mechanisms associated with cancer development and progression, in order to pave the way for new personalized medicine approaches. Interestingly, abnormal levels of the DGK substrate, DAG, are involved in malignant cell transformation, as increased DAG levels induce tumor-promoting effects. Consequently, a decreased expression or activity of DGKs could lead to higher DAG levels that could promote malignant cell transformation (Figure 1) [17,35]. 

DGKζ activity may explain clearly how DGKs negatively regulate DAG in order to limit the transforming potential of DAG in cancer [37]. Following T cell receptor stimulation, DGKζ negatively regulates RasGRP1, an enzyme involved in cell proliferation. As such, excessive activation of the oncogenic protein Ras is observed in DGKζ-deficient lymphocytes upon T cell receptor stimulation and this correlates with high DAG levels. Even DGKα seems to modulate RasGRP1 and its deletion induces hyperproliferation in T cells [58,65]. Besides, high levels of DGKα expression correlate positively with lung cancer patient survival [95], whereas in HCC, the downregulation of DGKα inhibits cell proliferation and metastasis [27]. Hence, DGKs act as both tumor suppressors and tumor promoters in cancer but this is cancer cell type-dependent.

All these reports demonstrate that DGK signaling may be essential in cancer development and progression. As such, this part of the review highlights the impact of DGKs in the regulation of cell growth, proliferation, and metastasis in cancer.

### 4.1. Cell Growth and Proliferation in Cancer

Cell growth and proliferation are essential factors in cancer development and progression. These processes have been shown to be regulated by the altered expression and/or activity of cell cycle-related proteins in cancer cells [17,96]. Several reports demonstrated the involvement of the DGK family in cell cycle regulation. For example, DGKs α and ζ play contrasting roles in regulating the cell cycle. DGKζ inhibited the progression of cells from G1 to S phase of the cell cycle [62], while DGKα-induced PA was required for IL-2 mediated progression of cells to the S phase [54]. DGKζ is a negative regulator of cell cycle progression in C2C12 mouse myoblasts: DGKζ overexpression blocked cells at the G1 phase of the cell cycle via its interaction with the Retinoblastoma protein (pRb) which is a tumor suppressor and a cell cycle regulator, and DGKζ downregulation increased the number of cells at both S and G2/M phases of the cell cycle [97]. Interestingly, DGKζ is highly expressed in patient-derived acute myeloid leukemia cells and the knockdown of DGKζ in HL-60 promyelocytic cells induces a cell cycle arrest at the G2M checkpoint, inhibiting cell proliferation while increasing apoptosis (Table 2) [98]. In addition, DGKζ inhibition in U251 and U87 glioblastoma cells caused a marked decrease in cyclin D1 (CCND1) protein expression, which led to an arrest of cells at the G0/G1 phase [99]. Furthermore, major regulators of cancer cell growth and proliferation, such as the phosphorylated forms of Akt and mTOR, were also decreased, resulting in a significant reduction of cell proliferation in DGKζ knockdown cells compared to control cells. The authors also showed in an in vivo model that the tumorigenic capability of glioblastoma cells was reduced when DGKζ expression was decreased. Hence, DGKζ inhibition may confer advantages to glioblastoma patients [99]. 

In other cancer cells, such as K562 human erythroleukemia cells, DGKα can modulate cell cycle progression by influencing the phosphorylated status of pRb, which subsequently induces cell cycle arrest by impairing the G1/S transition [100]. In HCC cells, DGKα knockdown significantly suppresses cell proliferation, whereas overexpressing wildtype DGKα but not the kinase-dead mutant in the same cells significantly enhances proliferation. Similar results were obtained in HCC xenograft model experiments, where DGKα regulates cell proliferation via activation of the MAPK pathway. Specifically, DGKα downregulation impaired mitogen-activated protein kinase (MEK) and extracellular signal-regulated kinase (ERK) phosphorylation, both of which are crucial in the regulation of cell growth and migration [27]. Moreover, a novel DGKα specific inhibitor CU-3, which was successfully obtained after a high-throughput screening of about 9600 chemical compounds, induced apoptosis in HepG2 HCC cells and HeLa cervical cancer cells, while simultaneously enhancing immune response by promoting IL-2 production [101]. Consistent with these data, it was also reported that silencing or inhibiting DGKα activity with short interfering RNA (siRNA) or small-molecule inhibitor R59022 caused increased death of glioblastoma and melanoma cells by interrupting essential oncogenic pathways [29]. DGKα knockdown decreased both total and phosphorylated forms of mTOR, hypoxia-inducible factor 1-alpha (HIF1α), c-Myc levels, and phosphorylation of Akt in glioblastoma cells. Xenograft experiments also demonstrated that DGKα knockdown and inhibition affects tumor growth, angiogenesis, and survival of mice with intracranial and subcutaneous tumors. Intriguingly, knockdown of DGKα in non-cancerous cells, such as astrocytes and fibroblasts, showed no form of cytotoxicity as revealed in both glioblastoma and melanoma cells. Hence, small-molecule inhibition of DGKα is selectively toxic to human cancer cells but not normal human cells, thus making DGKα inhibition a promising therapeutic target [29].

Several studies demonstrated an active role of DGKα also in Src oncogenic functions [8,28]. Src is a regulator of mitogenic and survival signaling pathways that are downstream of receptor and non-receptor tyrosine kinases, such as the vascular endothelial growth factor receptor (VEGFR), human epidermal growth factor receptor-2 (HER2) and focal adhesion kinase (FAK), which are often aberrantly expressed in colon, breast, and pancreatic cancer [8]. Using 3D colon and breast cancer cell cultures, it was demonstrated that DGKα is essential in cell growth and survival by promoting the stabilization of Src activation. Importantly, DGKα enzymatic activity is necessary for Src activation. Pharmacological or genetic DGKα silencing restricted tumor growth in vivo, thus confirming the function of DGKα in malignant transformation [28].

Furthermore, DGK is involved in the major biological features of the transformed phenotype of Kaposi’s sarcoma (KS) cells, where DGK is essential for cell proliferation and DGK inhibitors could be promising for therapy [104]. Indeed, the DGK pharmacological inhibitor R59949 strongly reduces hepatocyte growth factor (HGF)-induced KS proliferation and anchorage-independent growth without affecting cell survival or the classical Akt and MAPK pathways, which are usually implicated in KS.

On the other hand, further studies showed that CHO-K1 ovary cells expressing the kinase negative mutant of DGKγ exhibited a larger size, slower growth rate, and an extended S phase, suggesting that the increase of cell size was induced by protein synthesis during the extended S phase and that DGKγ regulates cell cycle [61]. Even though the activities of DGKs in cancer seem to support tumor-promoting roles, there is evidence that DGKs can also support tumor suppressor activities [102]. For instance, DGKγ expression is downregulated in HCC tumor tissues and colorectal cancer (CRC) cell lines when compared to non-tumor control tissues, and this correlates with poor clinical outcomes [102]. Interestingly, DGKγ downregulation in HCC is due to epigenetic mutations induced by histone H3 and H4 deacetylation. In addition, an analysis of methylation of the CpG islands of DGK promoter genes in CRC cells revealed that DGKγ is hypermethylated in CRC cells but not in normal colonic tissue, and this corresponds with reduced DGKγ expression in CRC cell lines compared to control cells [26]. However, both constitutively active and kinase-dead DGKγ mutants induced inhibitory effects on CRC cell proliferation [26]. Notably, the ectopic expression of DGKγ in HCC cells decreased cell growth by downregulating glucose transporter 1 (GLUT1) expression and inhibiting cell glycolysis. In fact, GLUT1 expression is high in HCC and promotes tumorigenicity, therefore DGKγ plays tumor suppressor roles in HCC by lowering GLUT1 levels [102]. 

DGKε activity can also regulate the Ras/RAF/MEK/ERK signaling in cervical cancer cell line models [33]. This pathway plays pivotal roles in the regulation of cell proliferation, survival, and differentiation. The study showed that siRNA downregulation of DGKε impairs the epidermal growth factor (EGF)-activated Ras/RAF/MEK/ERK signaling cascade in HeLa cells. However, the mechanism through which DGKε regulates this pathway is still unknown [33]. Additionally, the potential of DGKη to regulate MAPK signaling, which is a downstream target of epidermal growth factor receptor (EGFR), led a group to study the oncogenic effects of DGKη in lung cancer, which is often characterized by mutations in EGFR and KRAS. The authors reported that silencing DGKη in lung cancer models, characterized by EGFR and KRAS mutations, reduced cancer cell growth while enhancing the cells’ sensitivity to EGFR inhibitor Afatinib [103].

### 4.2. Cell Migration, Invasiveness, and Metastasis

The motility and invasion of cancer cells from the primary tumor to a distant organ is an essential step in tumor metastasis. This event requires chemotactic migration of cancer cells and crossing of extracellular matrix barriers that surround the tumor [105]. As previously stated, DGKγ plays tumor-suppressive roles in CRC. The ectopic expression of wildtype, as well as kinase active and inactive mutant forms of DGKγ, restricts cell migration and invasion in CRC cells by inhibiting Rac1 activity [26]. Rac1 is a member of the Rho GTPases, which are small GTP-binding proteins that regulate cytoskeletal dynamics and activate essential protein kinases involved in Epithelial to Mesenchymal Transition (EMT) [106]. EMT involves the reprogramming of epithelial cells into mesenchymal cells, leading to morphological changes, specifically more elongated and spindle-like forms with increased migratory and invasive properties [107]. Notably, Rac1 is highly expressed in different stages of colorectal tumors. Its activity in CRC tissues positively correlates with poor prognosis of CRC patients by promoting EMT-mediated invasion of CRC cells via the activation of the signal transducers and activators of transcription 3 (STAT3) pathway [106]. Therefore, it would be interesting to elucidate the mechanisms associated with DGKγ-mediated inhibition of Rac1 activity for potential CRC therapy. DGKγ also plays tumor suppressor roles in HCC cells by reducing cell migration when DGKγ is overexpressed [102]. Conversely, DGKα is highly expressed in HCC and promotes tumorigenicity [27]. In fact, knockdown of DGKα suppresses cell migration by impairing the Ras/RAF/MEK/ERK pathway in HCC cells. The Ras/RAF/MEK/ERK pathway is indeed frequently deregulated in HCC and the activation of this pathway is significantly involved in cancer cell invasion [27]. In fact, ERK signaling is a critical mediator of cell migration, although it is also a classic mediator of cell growth, proliferation, and differentiation. ERK activates several proteins that regulate cell-matrix adhesion, cell protrusion, and retraction, all of which are essential processes recognized during cell motility [108]. Moreover, ERK controls EMT-regulated cell migration through Rac1/Fox01 activation [107]. DGKα activity has also been reported to be a key factor in the migratory and invasive responses induced by several growth factors, including HGF and vascular endothelial growth factor (VEGF) in endothelial, epithelial, and leukemic cells [109,110,111,112]. In line with the potential to regulate migration in endothelial cells by DGKα, a study employing both DGKα specific siRNA and/or DGK pharmacological inhibitor R59949 demonstrated that DGKα activity is a key regulator of migration in Hec-1A endometrial cancer cell line [112]. Inhibition of DGKα indeed reduced cell migration towards estrogen chemoattractant as well as abolished ruffle formation in Hec-1A cells [112]. In addition, DGKα promotes invasive migration in H1299 lung cancer cells and A2780 ovarian carcinoma cells by controlling Rab coupling protein (RCP)-driven integrin trafficking [113]. Furthermore, R59949 significantly reduced HGF-induced motility in KS cell lines with limited effects on cell adhesion and spreading [104], but it did not affect MAPK and Akt signaling pathways.

The downstream product of DGKs, PA has been associated with the receptor tyrosine kinase (RTK) signaling, which is an upstream regulator of the Ras/RAF/MEK/ERK cascade [114]. Another study attributed a potential role of PA in regulating tumor metastasis due to its ability to induce the secretion of Type 1 matrix metalloproteases (MMP1), enzymes able to promote metastasis [115]. However, these studies refer to PA generated by phospholipase D (PLD). Hence, it would be important to understand whether PA generated by DGKs performs the same functions. Interestingly, the application of nanomolar concentrations of PA increased cell migration in invasive MDA-MB-231 human breast cancer cells but had no effect on non-neoplastic control cells. Moreover, applying *Clostridium difficile* Toxin B to the PA-treated MDA-MB-231 breast cancer cells inhibited Rho activity and was followed by a marked decrease in cell migration [116]. These data strengthen the link between DGK/PA and Rho GTPases in cytoskeletal organization and subsequent cell migration. In addition, PA may be central in the regulation of cell motility by controlling the activity of type I PIP5K isozymes and PtdIns(4,5)P_2_ [117]. In fact, PA stimulates PIP5K, that participates in actin reorganization by generating PtdIns(4,5)P_2_, which is a primary regulator of cytoskeletal organization, so that PA signaling is also critical in PtdIns(4,5)P_2_ resynthesis [117]. 

A study reported that DGKζ deficiency in fibroblast cells induces a reduction in Rac1 and RhoA activation, as well as a significant reduction in cell migration [118]. Considering this finding, the authors extended their study by elucidating the impact of DGKζ signaling in CRC metastasis [30]. In tumor-derived CRC cell lines, knocking down DGKζ expression produced similar results as those seen in fibroblasts. A significant decrease in Rac1 and RhoA activity in DGKζ knockdown CRC cells was also observed and was followed by a decrease in cell invasion. Concomitantly, DGKζ depletion decreased the invasiveness of prostate cancer and metastatic breast cancer cells [30]. Thus, opposite to the tumor-suppressive roles of DGKγ as described above, DGKζ may also promote tumorigenesis by potentiating cell invasion and migration in several cancer types by regulating Rac1 and RhoA activity. This may be due to the fact that coordinated events between Rac1 and RhoA are necessary for effective migration in cancer metastasis. Indeed, RhoA is involved in the maintenance of actin stress fibers and focal adhesions, while Rac1 regulates the generation of lamellipodia, membrane ruffles formation, and Cdc42 signaling in filopodia production [119]. 

As for other DGKs, such as DGKδ and DGKι, there are a few reports demonstrating their participation in the development and progression of cancer. Downregulation of DGKδ in cervical and lung adenocarcinoma cell line models induced a downregulation of Akt activity, leading to a decrease in cell migration and proliferation. Moreover, DGKδ can control Akt activity through pleckstrin homology domain leucine-rich repeat protein phosphatase 2 (PHLPP2) [32]. Epigenetic studies have also revealed that DGKι may be methylated in cancer, including glioblastoma and HCC [34,35], while it is still unknown whether DGKι mutations may produce effects directly involved in metastasis of these cancer types.

## 5. Targeting DGKs in Cancer Therapies

The development of effective therapies to fight cancer continues to be one of the major challenges in modern medicine. Chemotherapy and radiotherapy are somehow successful, but these approaches are non-specific and often lead to short- or long-term adverse effects which usually affect quality of life [7]. Recently, exploring immune-based therapeutic systems, such as blockage of immune checkpoints or adoptive cell transfer (ACT) that stimulate antitumor immunity by targeting and attacking tumor cells, seems promising because of its specificity. The starting point of this immune response is represented by chimeric antigen receptor (CAR)-T cells [120]. Interestingly, incoming reports suggest that targeting DGK activity could be a strong approach to reinforce the anti-tumor functions of T cells [7].

Currently, DGKα holds much promise in cancer therapy [7,29], as its inhibition presents toxicity in various cancer, but not normal human cells [29]. For instance, in T cells, the inhibition of DGKα activity may generate a simultaneous response of reinforcing T cell attack on tumor cells, while directly inhibiting tumor growth [7]. 

The inhibition of DGKα by R59949 in KS and endometrial cancer cells leads to decreased cell proliferation, growth, and migration [104,112]. On the other hand, using the small molecule inhibitor of DGKs R59022, DGKα was inhibited in glioblastoma, cervical cancer, melanoma, and breast cancer cell lines. In these cells, the percentage of cell death was increased compared to control normal fibroblasts and astrocytes [29]. Indeed, in glioblastoma in vivo tumor models, the same authors showed that DGKα inhibition decreases angiogenesis, tumor growth, and survival of mice with tumors [29]. Targeting DGKs may be important in cancer immunotherapy, as intratumoral injection of DGK knockout T-cells into U87MGvIII glioblastoma tumor models, obtained by CRISPR/Cas9, caused significant suppression of the tumors [25]. More importantly, this result was due to an enhancement in the effector functions of T-cells in the xenograft model. The authors also showed that the CRISPR/Cas9 generated DGK-knockout in CAR-T cells potentiates T-cell functions by increasing cluster of differentiation 3 (CD3) signaling. Consequently, the cells became resistant to immunosuppressive factors such as transforming growth factor-β (TGFβ) and prostaglandin E2, which are known mediators of cancer cell survival [25]. 

**Figure 2:**
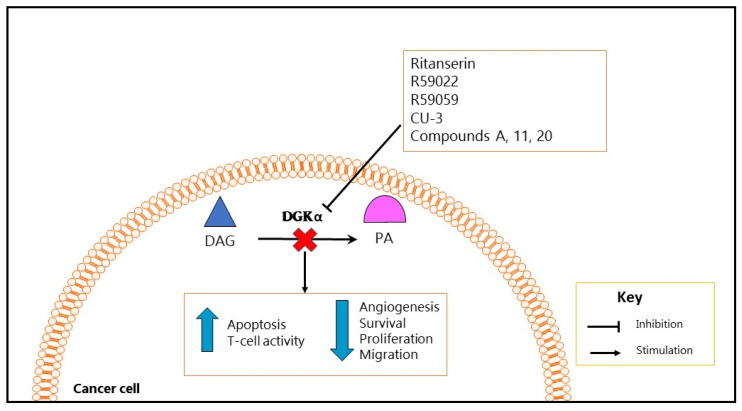
Functional effects produced by DGKα inhibition in cancer. The use of DGKα specific or pan inhibitors to inhibit DGKα activity in cancer cells seems to block cancer progression through the inhibition of cancer-promoting mechanisms, such as growth and survival. Intriguingly, inhibition of DGKα activity increases apoptosis in several cancer cell lines and in vivo tumor models, while enhancing T-cell activity or immune response.

Other studies tested CU-3, a DGK pharmacological inhibitor with a higher specificity for DGKα than R59949 and R59022, mainly due to its specific targeting of the ATP binding site in the catalytic domain of DGKα. This molecule induced apoptosis in several cancer cells, while enhancing immune response [101]. Similarly, compound A, which specifically inhibits type I DGKs and especially DGKα, induced apoptosis and reduced viability of melanoma and several other cancer cell lines [121]. In addition, two novel DGKα inhibitor compounds, namely 11 and 20 (with an IC_50_ of 1.6 and 1.8 µM respectively, thus representing the most potent DGKα inhibitors until now), decreased cell migration in cancer cells (Figure 2) [122].

On the other hand, Ritanserin, an established serotonin receptor inhibitor, has recently been identified as a DGKα inhibitor. Interestingly, it is more potent than R59022, although these two compounds differ structurally by just a single fluorine [123]. Ritanserin has already been shown to be well-tolerated and safe for human use in clinical trials, potentially paving the way to use it clinically as a DGKα inhibitor [123]. In fact, treatment of several cancer cells with Ritanserin has yielded similar results as other DGK inhibitors [7,124]. For example, the mesenchymal subtypes of lung and pancreatic carcinoma, as well as the mesenchymal subtype of glioblastoma, are sensitive to Ritanserin. Indeed, DGKα inhibition by Ritanserin induced cell death in glioblastoma stem cells and this was partially mediated by apoptosis [124]. Additionally, Ritanserin, as with other small molecule inhibitors of DGKα, also enhanced T cell signaling but failed to promote long-term T-cell activation [125]. 

## 6. Conclusions

All the reported studies highlight DGK signaling as a promising target for cancer therapy. However, more studies are needed to fully comprehend DGK specific roles in cancer development and progression. Due to the isoform-specific functions observed in different types of cancer cells and even subcellular sites, it would be crucial to fully understand how the specific DGK isoforms control downstream oncogenic signaling, as these pathways can regulate proliferation, growth, angiogenesis, immunity, and migration. To better understand DGK signaling it would also be essential to explore the potential crosstalk among the various DGK isoforms, the possible redundancy or compensatory functions of other isoforms during the inhibition of one or more DGK isoforms, as well as consider the DGK specific subcellular localization relevance. Moreover, the discovery of more potent DGK-specific isoform inhibitors may be useful to study the isoform-specific functions and develop new cancer targeted therapies. Future studies may also benefit from the combinatorial effect of DGK inhibitors and other standard cancer therapies, such as radiation and chemotherapy. Furthermore, since PA is involved in several cellular processes, the combination of both DGK inhibitors and inhibitors of PA-synthesizing enzymes may prove to be more beneficial in cancer therapy than when used individually. Finally, since recent reports demonstrated that DGKs may have a preference for specific DAG species, which are independent of PI turnover pathways, it would be strategic to further investigate the cellular signaling pathways associated with PA, produced by both PI independent and PI dependent pathways. 

## Figures and Tables

**Table 1:** Distribution of DGKs across distinct subcellular compartments.

DGK Isoforms	Subcellular Localization
*DGKα*	Plasma membrane, Cytosol, Nucleus
*DGKβ*	Cytoskeleton
*DGKγ*	Nucleus, Golgi, Cytosol
*DGKδ*	Plasma membrane, Endoplasmic reticulum, Endosomes
*DGKε*	Plasma membrane, Endoplasmic reticulum
*DGKζ*	Nuclear speckles, Plasma membrane, Cytosol, Cytoskeleton
*DGKθ*	Nuclear speckles, Plasma membrane, Cytosol
*DGKι*	Nucleus, Cytosol
*DGKκ*	Plasma membrane
*DGKη*	Endosomes

**Table 2:** Functional effects on cancer upon the downregulation of DGKs.

DGK Isoform	Cancer Type	Effects Caused by Downregulating DGKs
**DGKα**	Glioblastoma	Decreases Akt/mTOR, HIF1α, c-Myc activity [29]
	HCC	Inhibits the Ras/RAF/MEK/ERK pathway [27]
	Acute Myeloid Leukemia	Impairs pRb signaling [100]
	Cervical cancer, HCC	Promotes IL-2 signaling [101]
	Colon, Breast cancer	Impairs Src activity [28]
**DGKδ**	Cervical cancer, Lung adenocarcinoma	Suppresses Akt activity [32]
**DGKε**	Cervical cancer	Impairs Ras/RAF/MEK/ERK signaling [33]
**DGKγ**	HCC	Inhibition of GLUT1 expression and glycolysis [102]
	Colorectal cancer	Inhibits Rac1 activity [26]
**DGKζ**	Glioblastoma	Suppresses Cyclin D1 expression [99] Decreases Akt/mTOR activity [99] Increases CD3 expression to enhance T-cell functions [25]
	Colorectal cancer	Decreases Rac1 and RhoA activity [30]
	Acute Myeloid Leukemia	Induces cell cycle arrest at G2M, Inhibits cell proliferation and increases apoptosis [98]
**DGKη**	Lung cancer	Impairs MAPK signaling [103]

## Abbreviations

3D	Three-dimensional
Ca^2+^	Calcium
ACT	Adoptive cell transfer
AML	Acute myeloid leukemia
CAR	Chimeric antigen receptor
CCND1	Cyclin D1
CD3	Cluster of differentiation 3
CRC	Colorectal cancer
DAG	Diacylglycerol
DGKs	Diacylglycerol kinases
EGF	Epidermal growth factor
EGFR	Epidermal growth factor receptor
EMT	Epithelial to mesenchymal transition
ERK	Extracellular signal-regulated kinase
FAK	Focal adhesion kinase
GAP	GTPase-activating protein
GLUT1	Glucose transporter 1
HCC	Hepatocellular carcinoma
HER2	Human epidermal growth factor receptor-2
HGF	Hepatocyte growth factor
HIF1α	Hypoxia-inducible factor 1-alpha
IL-2	Interleukin 2
InsP3R	Inositol trisphosphate receptor
KS	Kaposi’s sarcoma
MAPK	Mitogen activated protein kinase
MARCKS	Myristoylated alanine rich kinase substrate
MEK	Mitogen-activated protein kinase
MMP1	Type 1 matrix metalloproteases
mTOR	Mammalian target of rapamycin
NLS	Nuclear localization sequence
PA	Phosphatidic acid
PHLPP2	Pleckstrin homology domain leucin-rich repeat protein phosphatase 2
PI	Phosphoinositide
PI3K	Phosphoinositide 3-kinase
PIP5K	Phosphatidylinositol-4-phosphate 5-kinase
PKC	Protein kinase C
PLC	Phospholipases C
PLD	Phospholipase D
pRb	Retinoblastoma protein
PS	Phosphatidylserine
PtdIns(4,5)P2	Phosphatidylinositol 4,5 biphosphate
RCP Raf-1	Rab-coupling protein Rapidly accelerated fibrosarcoma-1
RasGRP	Rat sarcoma virus guanyl nucleotide-releasing protein
RTK	Receptor tyrosine kinase
SAM	Sterile α motif
siRNA	Short interfering RNA
SMSr	Sphingomyelin synthase related protein
STAT3	Signal transducers and activators of transcription 3
TGFβ	Transforming growth factor β
Unc-13	Mammalian uncoordinated 13
VEGF	Vascular endothelial growth factor
VEGFR	Vascular endothelial growth factor receptor
